# Automatically recognizing strategic cooperative behaviors in various situations of a team sport

**DOI:** 10.1371/journal.pone.0209247

**Published:** 2018-12-18

**Authors:** Motokazu Hojo, Keisuke Fujii, Yuki Inaba, Yoichi Motoyasu, Yoshinobu Kawahara

**Affiliations:** 1 RIKEN Center for Advanced Intelligence Project, Osaka, Japan; 2 Japanese Institute of Sports Sciences, Tokyo, Japan; 3 Tokuyama University, Yamaguchi, Japan; 4 The Institute of Scientific and Industrial Research, Osaka University, Osaka, Japan; Middlesex University, UNITED KINGDOM

## Abstract

Understanding multi-agent cooperative behavior is challenging in various scientific and engineering domains. In some cases, such as team sports, many cooperative behaviors can be visually categorized and labeled manually by experts. However, these actions which are manually categorized with the same label based on its function have low spatiotemporal similarity. In other words, it is difficult to find similar and different structures of the motions with the same and different labels, respectively. Here, we propose an automatic recognition system for strategic cooperative plays, which are the minimal, basic, and diverse plays in a ball game. Using player’s moving distance, geometric information, and distances among players, the proposed method accurately discriminated not only the cooperative plays in a primary area, i.e., near the ball, but also those distant from a primary area. We also propose a method to classify more detailed types of cooperative plays in various situations. The proposed framework, which sheds light on inconspicuous players to play important roles, could have a potential to detect well-defined and labeled cooperative behaviors.

## Introduction

Groups of social organisms, such as fishes, birds, and humans including people performing sports, cooperate to achieve common goals that could not be achieved by an individual. This type of cooperation has been referred to as collective intelligence [1], team synergy [2], or teamwork. This multi-agent interactions have attracted attention in several scientific and engineering fields, such as biology [3, 4], human behavioral science [1, 5, 6], including modeling [7, 8], and social psychology [9–11], and robotics [12, 13] including simulated agents [14, 15]. Studies into artificial multi-agents [12–15] have primarily focused on methods to realize the desired collective movements (i.e., a forward problem). However, multi-agent systems, particularly groups of living organisms, show various and intelligent movements wherein the dominance law is sometimes unclear [16, 17]. If we can classify the complex and functional behavior from data (i.e., an inverse problem), this may promote the understanding of functions of strategic cooperation. Meanwhile, studies into biological collective motion have primarily focused on global statistical properties, which aggregate information of individual agent in a group, such as group polarization and angular momentum in fish-schooling behavior [4]. However, in functional and complex collective motions, such as human groups including team sports, the cooperation often occurs locally and often changes their rules and objectives in a time-dependent manner according to various situations [16, 17]. Therefore, the large variance of behaviors even within the same functional formations makes it difficult to find similar and different motion structures using the same and different labels, respectively. Human groups performing sports, one of the representative research subjects above, have been recently studied to clarify the relationship between collective motion and an objective such as team-play [16, 18, 19] and achieving a score [20, 21]. These studies employed video-based position tracking systems, which measure all players and ball positions in several sports, such as football [22] and basketball [23].

In team sports, automatically detecting how players cooperate with teammates in various situations is a challenging task because of the large variance in behaviors with the same functions. Most applications of video-based position tracking systems are individual analyses, such as fitness indicators (e.g., moving distance or velocity [24]) and an expectation value of an offensive player scoring [25]. However, in practice with respect to the cooperative plays, from a long time ago, they are visually detected and recorded as well as scores. If we can utilize positional data obtained from tracking systems, an automatic recognition system for cooperative plays can be derived. However, cooperative plays with the same label (i.e., objective) sometimes differ structurally (i.e., temporally and/or spatially) even within the same plays because players must react to how teammates and opponents play (S1 Fig).

Generally, automatic classification methods for labeled data employ supervised learning. For labeled cooperative plays, previous studies have employed support vector machines (SVM) and logistic regression to classify offensive [18] and defensive plays [19] in specific situations, respectively. These studies segmented the interval of the candidate of the plays and extracted a hand-crafted feature vector to input to a classifier, such as the distances between players. These studies focused on specific cooperative plays in a primary area (i.e., near the ball, which is referred to as “on-ball play”); thus, effective segmentation and feature extraction can be obtained by focusing on the ball movement. However, cooperative plays frequently occur distant from the ball (referred to as “off-ball play”). Such plays have been recently analyzed as effective cooperative behavior such as in football [26, 27] primarily from the perspective of statistical dynamics (i.e., not analyzed labeled plays).

Among many team sports and their plays, the basic and minimal strategic cooperative play in basketball called ‘screen-play’ is a good example for an automatic recognition system of strategic cooperative plays. This is because they are well-defined and widely used plays that occur in various situations and locations, including off-ball situations. Screen-play is the play in which an offense player (called a ‘screener’) is standing on course of defense player like a wall and prevents the defense movement against another offense player (called a “user”) in a legal way. Previous studies classified the limited screen-plays for the player with the ball (called “on-ball screen-play”) [18, 19]. However, screen-plays distant from the ball (called “off-ball screen-play”), which can occur anytime, anywhere, and sometimes simultaneously, involve more diverse behaviors than on-ball screen-plays. Thus, classification including off-ball screen-plays as a representative example of labeled off-ball cooperative plays would be more difficult to detect automatically than plays near the ball, and may be meaningful for classifying labeled cooperative behavior in many scientific fields. In this study, therefore, we attempt to automatically classify all types of screen-plays.

However, there are two main problems to create a classifier to detect on- and off-ball cooperative plays. The first problem is diversity of types of off-ball cooperative plays as explained above. In actual games, players select more diverse types of screen-plays in various contexts than those of on-ball plays, such as coach’s instruction and adaptation to a particular situation. Therefore, we first visually categorize all types of screen-plays in a controlled experiment in a half-court game to reflect coaches’ instruction. The second problem is that a large amount of labeled data (i.e., numbers of screen-plays) is required for automatic classification with supervised learning. Moreover, some types of screen-plays dominantly occur but others seldom happen. Thus, in addition to using a larger amount of data obtained by an automatic tracking system [22, 23] in a real game, we first propose a method to detect the basic categories of screen-plays: on-ball and off-ball screen-plays (both can occur in sufficient amounts). Next, with the assumption of an accurate off-ball screen-play classifier, we propose a method to classify various types of off-ball screen-plays (see Results: on-ball screen-plays have fewer variations than off-ball screen-plays).

Therefore, in this study, we propose a classification method for labeled cooperative behaviors in a multi-agent system, i.e., a minimal strategic cooperative play in a real sport. We first examine the frequencies of different types of screen-plays in a controlled experiment. Then, we propose a method to automatically detect cooperative plays with a larger amount of data from an actual game. These methods are expected to shed light on inconspicuous players (i.e., players who are distant from the ball) to play important roles. A potential of the proposed framework to detect general well-defined labeled cooperative behavior is discussed in the Discussion section. Note that, although competitive interactions has been analysed often such as in 1-vs-1 experiments (e.g., [28, 29]) in sports science, this type of interaction regarding many players should be investigated from the data obtained from actual 5-vs-5 settings.

## Methods

### Participants and measurements in a controlled experiment

Ten males from a top-level university basketball team (top 2 at intercollege championship) in Japan (age = 19.5 ± 0.5 years, experience = 10.7 ± 2.4 years [mean ± SD]) participated in this study. The players provided written informed consent to participate in this study. The experimental procedures were conducted in accordance with the Declaration of Helsinki and this study was approved by the Local Ethics Committee of the Research Center of Health Physical Fitness and Sports, Nagoya University. One of the authors (Keisuke Fujii) belonged to this organization before.

### Design in a controlled experiment

The players were divided into two teams (team A and B) and played a five-on-five half-court (14 m ×15 m) basketball game alternately as the offensive team to shoot the ball within 20 s and then as the defensive team. Regarding the players’ position, two guards, two forwards, and one center for each team participated in this study. All of the team strategies at each attack, which the team usually used in actual games and had many screen-plays, were pre-determined by the coach and shared among the players. We analyzed 55 attacks (the number of the attacks was controlled). We obtained 140 actual screen-plays from these attacks, which was considered to be sufficient to understand a global tendency, according to the previous study [16]. For the measurements, the three-dimensional (3D) coordinates of the landmark points were acquired using a 3D optical motion capture system with six cameras operating at 100 Hz (OptiTrack Prime W17, NaturalPoint, USA). Camera positions (or other detailed experimental setup) were shown in the previous study [16]. Synchronization of the cameras was performed with the hub of the camera systems. Six cameras were the minimum number which can capture the half-court. Detail protocols are given in S1 Methods.

### Participants and measurement in a real game

The positional data of players and the ball (25 frames per second) was recorded and calculated by using STATS SportVU system in the real international games held in 2015. The positional data contained the XY position of each player on the court and the XYZ coordinates of the ball. We could not control the number of attacks in a real game so that we chose the time interval to obtain accurate data. To obtain accurate data, 94 minutes of play in which the two teams scored 316 points were analyzed.

### Data segmentation

Prior to data segmentation, we used an automatic individual play-detection system such as shot using the positional data. In addition, for our analyses and to obtain label information, all types of screens were categorized visually (for detail, see S1 Methods). In data segmentation, segmented 2038 positional datasets (called ‘actions’), in which an offense player move to a defense player and two offenses who might use the screen and their defenses (shown in S2 Fig) were automatically detected. At least two attackers related to a screen-play; a screener and a user. A screener is defined as the attacker to set the screen. A user is defined as the attacker to use the screen to free from the defender. In legal screen-plays, a screener set the screen and then the user starts to move. An off-ball screen-play is a screen-play without relation to the ball directly. In this study, an off-ball screen-play was defined as a screen-play in which the candidate screener and user do not possess the ball when the distance was the shortest (minimum distance in S2 Fig), and all other actions were defined as on-ball screen-plays. First, all offense players were considered a candidate screener, and for each candidate, two other offense and three defense players were defined as candidate users and candidate defenses for screeners and users. Then, a signal that screen-play was likely to occur was defined if the players satisfied both of following two conditions: (1) the distance between a candidate screener and a candidate user-defender was less than 1.2 m, and (2) the user-defender was the closest player to the candidate user. A user-defender is defined as the defender mainly defending user. For the frame in which the distance between the screener and the user-defender was the least and before and after 13 frames, signals were defined (S2 Fig). We refer to the first and last of 13 frames as the start time and end time, respectively. Too short signals (less than three successive frames) were excluded from the analysis and temporary adjacent same actions were jointed. This is because in a legal screen-play, a screener sets the screen and then the user moves in which the distance keeps short in certain time.

The authors, who have experience playing basketball, labeled all actions whether the action had a screen-play or not. As mentioned below, actions can be categorized into two plays (on-ball/off-ball screen-plays) to compare classification performance in advance.

### Extracted feature vectors

We created and used the same feature vectors for all classifiers (two-class SVM to detect screen-plays and the two multi-class SVMs to classify the detail type of off-ball screen-plays: one-against-all and one-against-one methods). We first computed the feature vectors employed in a previous study [18] for comparison. These feature vectors were used to classify the on-ball screen-play only. They used six distances between the two of the screener, user, user-defender and the goal. For each distance, they computed five features: the minimum distance, the average and change in distance in the interval from the start time to the time the distance was minimum and from the time when the distance was minimum to the end. Thus, the feature vectors had 30 dimensions in total. In this study, the moving distance and geometric information of each player were also considered. The moving distances comprised the distances of four players (the screener, defense of the user, and two candidate users) from the start to the end of the action. The geometric information includes the area where screen-plays are set (S3a Fig), the distance from each of the four players to the ball, and the angle of three players (S3b Fig). The area where screen-plays are set was categorized into nine areas in S3a Fig based on the position of the screener at the minimum distance in S2 Fig. Screen-plays generally occur more frequently in the horizontal middle areas than corner and the right area far from the goal, thus we analyzed the narrow middle areas were in detail. The distance from each of the four players to the ball was calculated using the position at the minimum distance in S2 Fig. The angle of the three players was defined by the position of the user (two candidates), the screener, and user-defender, as shown in S3b Fig. Next, the distance between players employed in the previous study [18] was expanded and was calculated as nine distances (screener to the defense of the user, screener to each of two users, defense of the user to each of two users and the goal to the four players). We analyzed 13 frames (the minimum distance frame and the former and latter six frames) shown in S3a Fig. Five feature vectors (minimum amplitude, average rate of change and mean amplitude during the former and latter seven frames including minimum distance frame) [18] were calculated for each of the nine distances. The mean value was employed because it is less sensitive than the variance of the minimum and maximum values. Therefore, 45-dimensional feature vectors were employed in this study. In this analysis, information on the moving distance (13 dimensions) and the geometric information (60 dimensions) were added to or eliminated from the feature vectors to compare classification performance. Therefore, the feature vectors had 148 dimensions in total.

### Classification

In this study, we employed a two-class SVM to detect screen-plays and a multi-class SVM to classify the detailed types of off-ball screen-plays. SVM is a widely used for discriminative classification to find the optimal hyper-plane between two classes [30]. In this study, we employed a soft margin SVM using a Gaussian-kernel (see S1 Methods). However, two-class SVM cannot directly classify data among more than three classes. Multi-class SVM can solve the limitation. For multi-class classification, we employed the multi-class SVMs executing multiple two-class SVMs, with two strategies: one-against-all and one-against-one [31–33]. Previous studies have suggested that it might be difficult to claim that one strategy is always better because accuracy may depend on the given data and tasks [31–33]. The one-against-all method constructs *k* SVM models (*k* is the number of classes), where one class is labeled as positive and the remaining classes are labeled as negative. The one-against-one method constructs *k*(*k*-1)/2 SVM models, where one class is labeled as positive, another class is labeled as negative and all other classes are ignored. In this study, we primarily show the results of the one-against-all SVM because off-ball screen-plays only demonstrated four classes because two classes did not have sufficient numbers of samples and the numbers of samples varied (Table 1). For problems with fewer classes, such as digit recognition (e.g., 11 classes), the one-against-all strategy seems significantly more accurate; however, with more classes, an imbalance in the number of samples may cause inaccurate classification [33]. In this study, the imbalance problem occurred in both strategies (Table 1), and the classification problem only considered seven classes; thus, we primarily discuss the one-against-all results (details of the one-against-one SVM are shown in Supplemental Methods, Supplemental Results and S4 Fig).

**Table 1:** Types of screen-plays in experiments and real games. All types of screen-plays in this study are shown. Down screen, back screen, and pick dominated the offense in the controlled experiment but down screen, flare screen, cross screen, and pick dominated the offense in the real game. Experiment and real game indicate our two different datasets: a controlled experiment and real game, respectively.

	Screen Type	experiment	real game	Screen Characteristics
off—ball	Down screen	33	104	A screener usually sets a screen relatively near the basket ring, and the user uses it to move toward the passer (away from the ring).
	Flare screen	12	68	A screener usually sets a screen far from the basket ring, and the user uses it to move away from the passer and the ring.
	Pin screen	10	23	A screener usually sets a screen relatively near the basket ring and base line, and the user uses it to move toward the corner.
	Back screen	31	31	A screener sets a screen on the defender’s back side far from the basket ring, and the user uses it to move toward the ring.
	Flex screen	8	8	A screener sets a screen far from the basket ring, and the user uses it to move parallel to the baseline (change the court).
	Cross screen	13	51	A screener moves parallel to the baseline toward the user and sets the screen, and the user moves toward the screener’s past position (the two players exchange court side positions).
on—ball	Pick & Roll	24	140	A screener sets a screen for the user who has the ball (usually dribbling).
	H and off	11	42	A screener has the ball, makes a handoff pass, and sets the screen for the user.
sum		142	467	

For screen-play detection (two-class classification), we divided into 1218 actions (on-ball: 496 actions; off-ball: 722 actions) for training sets and 611 actions (on-ball: 249 actions; off-ball: 362 actions) for test sets from the real game dataset (repeated five times as explained below). To classify the detailed types of off-ball screen-plays, we divided into 372 screen-plays for the training set and 95 screen-plays for the test set. For cross validation to determine the constraint parameter of SVM which represents the model complexity, we divided training sets into (small) train and validation sets. We trained SVM classifiers using train data with 10-fold cross validation and evaluated them with test data only. This process was repeated five times with different test sets created randomly and the mean values were calculated for evaluation.

Receiver operating characteristic (ROC) graph is a very useful tool for visualizing and evaluating classifiers. They are able to provide a richer measure of classification performance than scalar measures such as accuracy and error rate. The area under the curve (AUC) (based on the ROC curve) is a common method to compare classifiers and has an important statistical property; the AUC of a classifier is equivalent to the probability that the classifier will rank a randomly chosen positive instance higher than a randomly chosen negative instance [34]. The AUC was calculated using the ROC curve to examine the relationship between the true positive rate and false positive rate. The recall was defined as the ratio of the sum of true positives and true negatives to the number of true positives (the true positive rate), and precision was defined as the ratio of the sum of true positives and true negatives to false positives.

The trade-off curve between recall and precision was created using the cumulative distribution function. To evaluate the trade-off, the F score was calculated as follows:

Fscore=((2×Recall×Precision))/((Recall+Precision)). (1)

All AUC values and F scores were measured as the median of five repeated test sets.

## Results

### Type of plays in experimental data and segmentation performance

**Fig 1:**
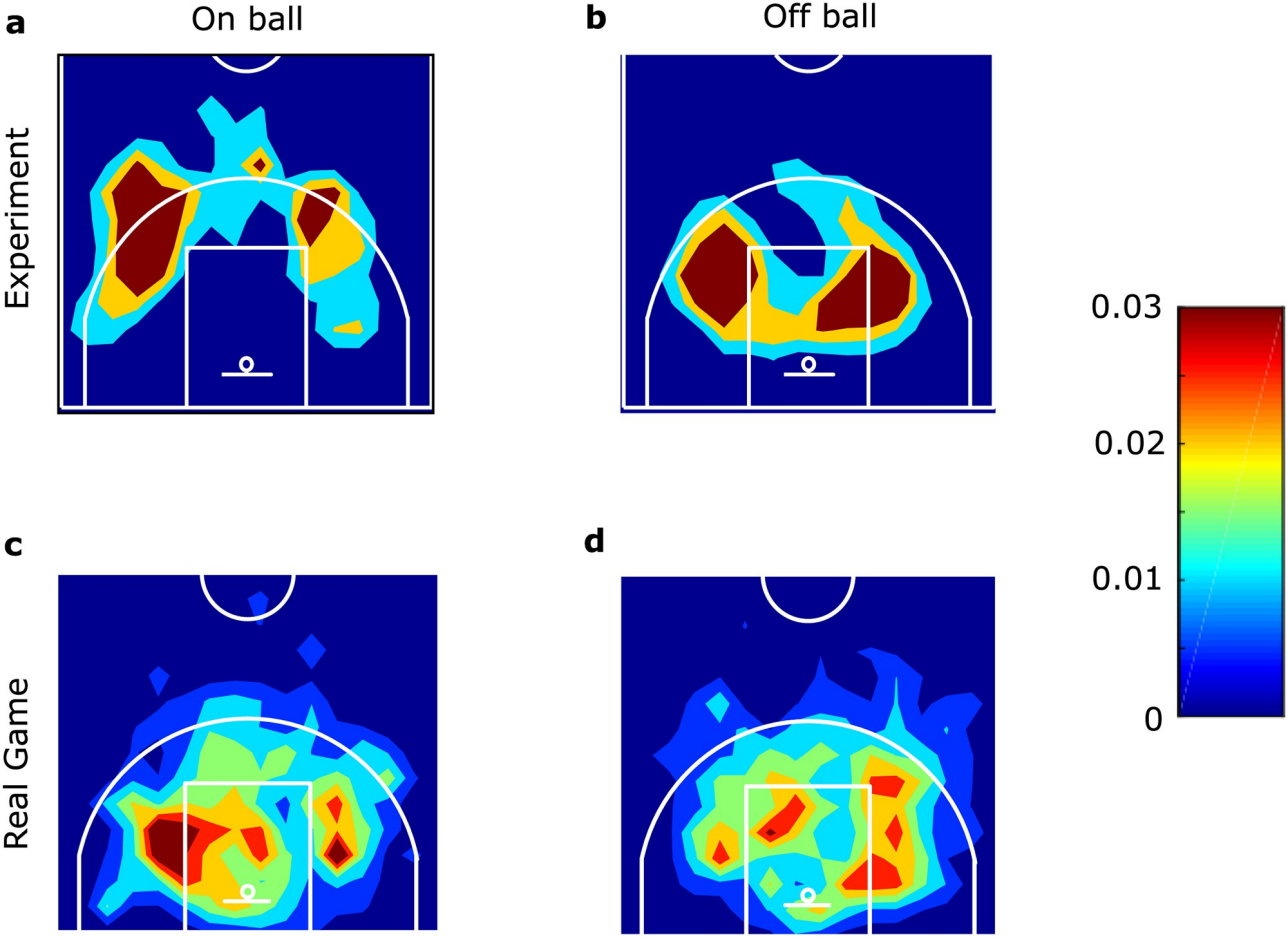
On- and off-ball screen-play movements. (a-d) Screener’s position in on- and off-ball screen-plays. (a-b) Results in the controlled experiment. (c-d) Results in the real games.

In the controlled experiment, we analyzed 55 attacks. We focused on screen-plays for the player with the ball (on-ball screen-plays: 35 plays) and those distant from the ball (off-ball screen-plays: 105 plays). S1 Fig and S1–S8 Movies (online) show representative examples of two on-ball and six off-ball screen-plays and their variations (descriptions are in Table 1). S1 Fig shows that plays with the same label are spatiotemporally different even within the same plays because players must react to their teammates and opponents. The heat maps of the histogram of screen-plays (Fig 1a and 1b) indicate that on-ball screen-plays were executed at a relatively greater distance from the goal than off-ball screen-plays probably because the defense player moved to keep the ball away from the goal. The frequencies of all types of screen-plays (Table 1, left) show that specific types of screen-plays (down, back, flare screens, and pick-and-roll screen-plays) dominated in all attacks. In data segmentation, we segmented the position tracking data series into segments (called ‘actions’) with short time intervals, including screen-play candidates, and labeled the actions as the screen-plays or not. 340 actions were detected as the candidate of screen-plays by automatic segmentation system in the controlled experiment. Note that labelling was performed for these actions. In these actions, 140 actions were labeled as screen-plays by the authors. However, four actions were labeled as screen-plays by authors but not detected as actions. In other words, the segmentation results demonstrate that 97.2% (140/144) of all on- and off-ball screen-plays were involved in actions; however, only 41.2% (140/340) of all actions contained on- and off-ball screen-plays.

### Automatic recognition of cooperative plays in a real game

Next, we attempted to create an automatic screen-play detection system using a greater amount of position tracking data from the real game than that used in the above experiment. In the real games, off-ball screen-plays occurred 26.4 times and on-ball screen-plays occurred 21.5 times every 10 minutes (in a real game, the definition of the attacks is difficult because players are not always in the same half court compared with the controlled experiment). Fig 1c and 1d show the frequency of the position where on- and off-ball screen-plays in the real game. The frequencies of all types of screen-plays examined in the experimental data are shown in Table 1 third and fourth columns. Compared to the frequency in the experimental data, the different but specific types of screen-plays (down screen, flare screen, back screen, and cross screen) dominated all attacks.

**Fig 2:**
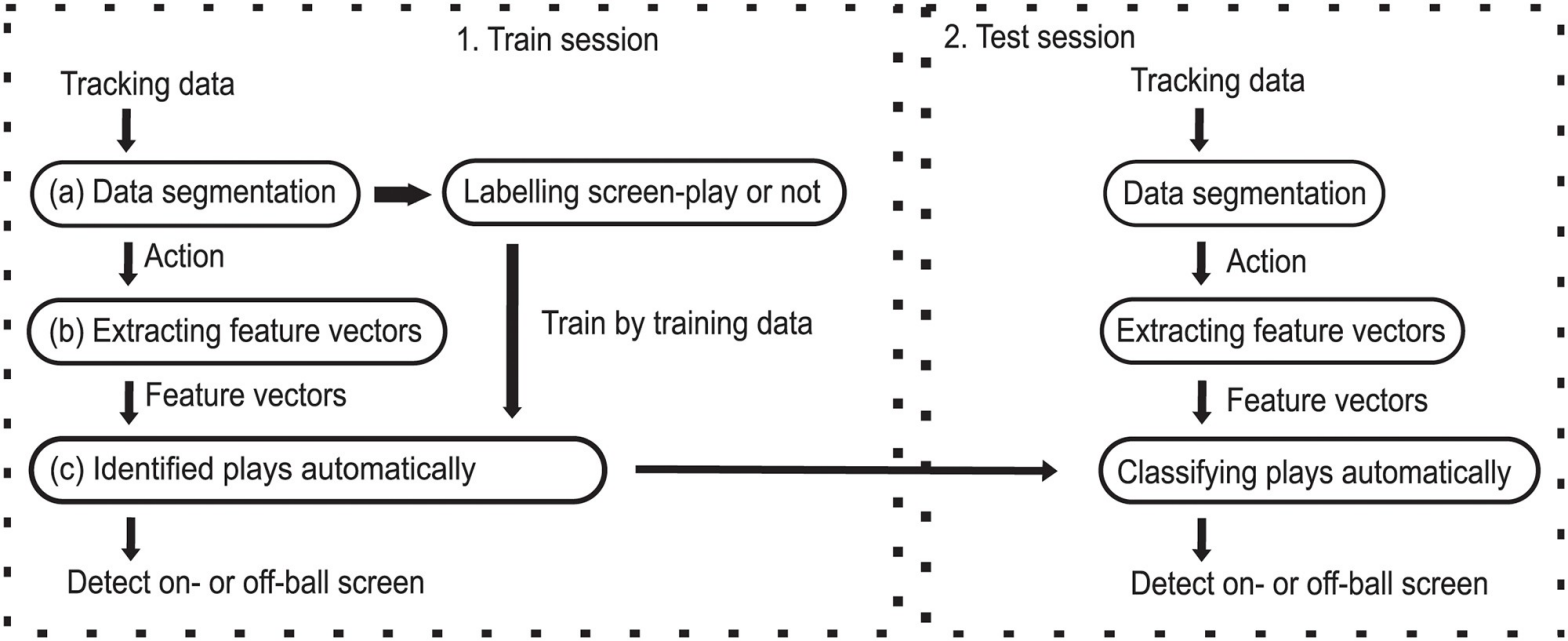
System flow diagram. In this study, the classification was composed of three steps: data segmentation, extracting feature vectors and classifying plays. In segmentation, data was segmented into actions. After segmenting train data, the actions were labeled as screen-play or not. From segmented data of actions, feature vectors were extracted. By using these labels and feature vectors, the classifier was trained in a training session. After the training session, test session was executed with test data.

Then, we propose an automatic detection system to detect on-ball and off-ball screen-plays from the position tracking data using a two-class SVM [30]. This system comprises three steps: data segmentation, feature extraction, and classification (Fig 2). Labelling was performed for 2038 actions in total and divided into a training set (on-ball: 496 actions, off-ball: 722 actions) and a test set (on-ball: 249 actions, off-ball: 362 actions). The segmentation performance for real games decreased to 22.9% (467/2038) compared with that for the experiment (41.2%). This was probably because of the diversity and variability of the plays in real games.

**Fig 3:**
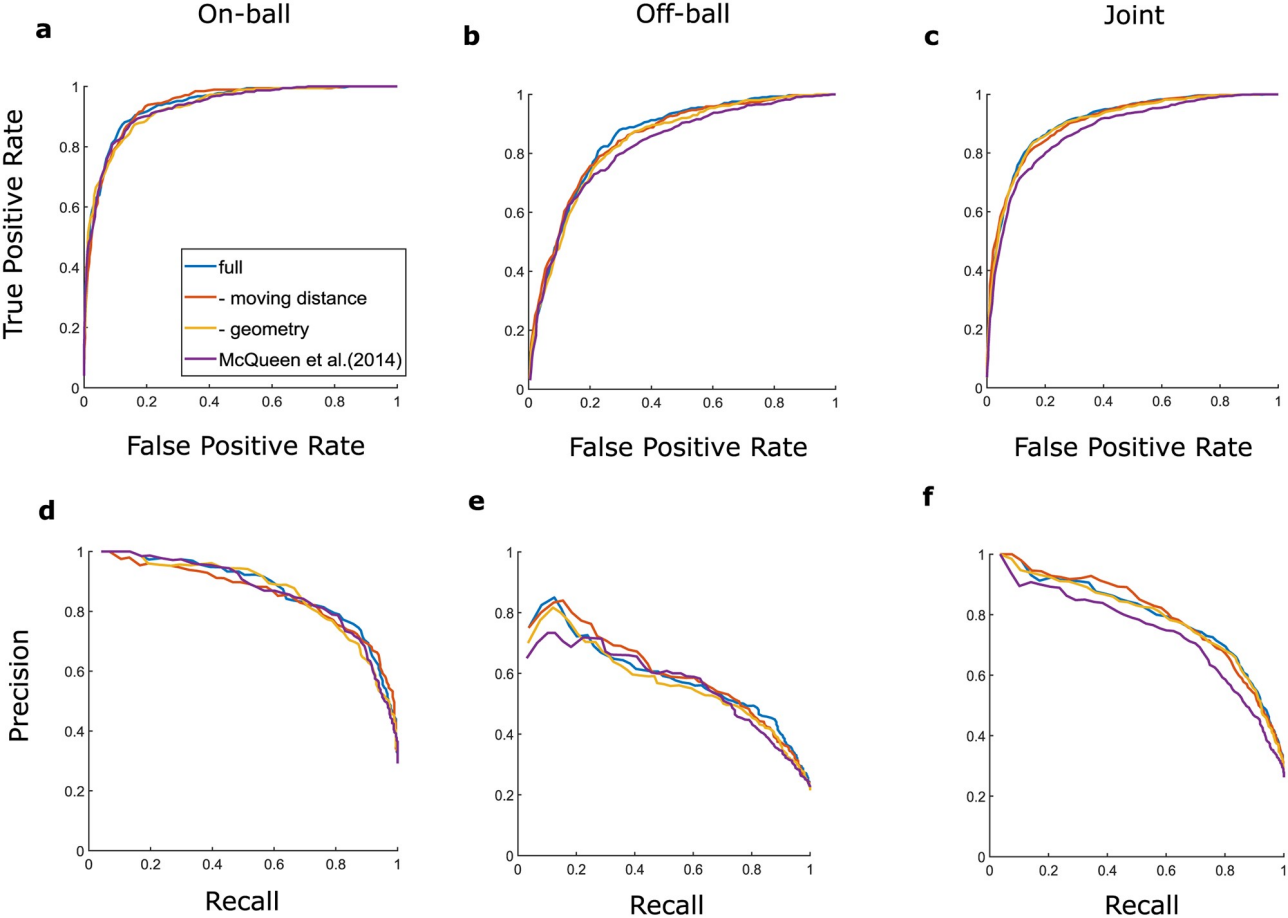
Receiver operating characteristic (ROC) and recall-precision trade-off curve in screen detection. ROC curves (top) and recall-precision trade-off (bottom) for on-ball (a and d), off-ball (b and e) and all screen-plays (c and f) are shown.

After segmentation, two-class SVM classifiers were trained separately and jointly for on- and off-ball screen-plays to compare classification performance. We refer to these classifiers as on-ball, off-ball and joint classifier, respectively. Note that in the former cases in which training separately, we automatically categorized on- and off-ball actions using the ball position data before classification. In the latter case, after classification where the training data was mixed, an evaluation was performed with both mixed test data and separated test data. The feature vectors used for the classifier were calculated from segmented data, such as distance among players and between each player and the ball, geometric information and individual moving distance. All plots include classification performance with and without moving distance and geometric information, as well as the result of the conventional method used by McQueen et al. (2014) [18]. In this study, we employed the ROC curve, AUC and F score to evaluate SVM classification performance. Classification performance using the ROC curve are shown in Fig 3. We used the AUC of the ROC curve as an indicator of classification performance. The mean AUC of the on-ball (Fig 3a), off-ball (Fig 3b) and joint classifier (Fig 3c) was 0.941, 0.855, and 0.911 (on-ball: 0.877, off-ball: 0.949), respectively (in the bracket we evaluated it with separate test data). Results of the comparison with the performance of the conventional method [18] show that the mean AUC of the on-ball, off-ball and joint classifier was 0.933, 0.825, and 0. 880 (on-ball: 0. 837, off-ball: 0.928), respectively. If the feature vectors did not include moving distance, the mean AUC was 0.940, 0.851, and 0.908 (on-ball: 0.871, off-ball: 0.948), respectively, and without geometric information, the mean AUC was 0.933, 0.840, and 0.906 (on-ball: 0.873, off-ball: 0.943), respectively. The results of AUC show that the separate classifier yielded better classification performance for on-ball screen-plays but joint classifier demonstrated better results for off-ball screen-plays. In summary, our method can effectively detect both on- and off-ball screen-plays.

**Fig 4:**
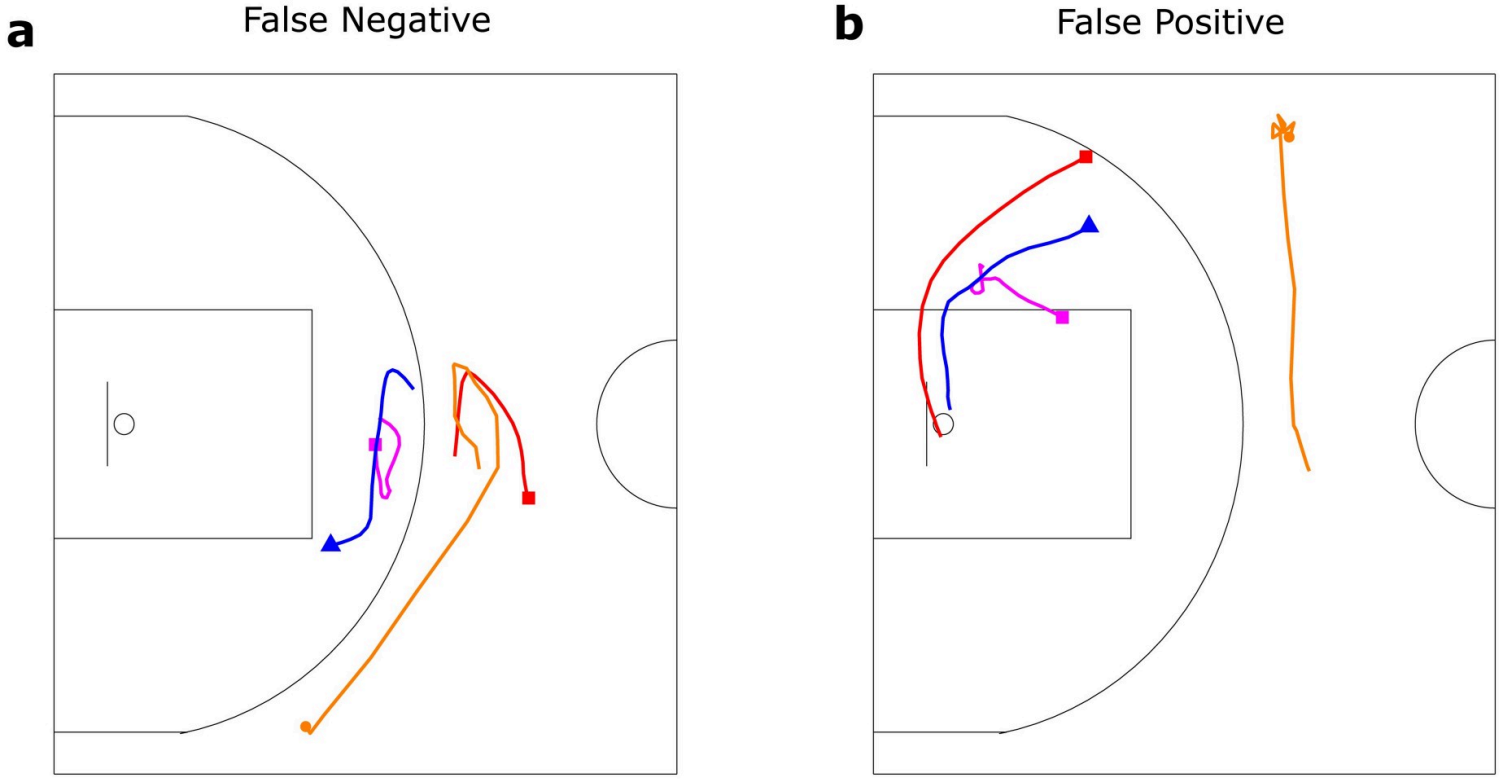
Screen detection misclassification. Examples of (a) false negative (miss) and (b) false positive (false-alarm) errors. Red, magenta, blue and orange lines represent the screener, user, defense of the user and the ball respectively. Triangles and circles represent their start positions. (a) Screener set a screen but the user moved far from screener so that this play was not classified as screen-play. (b) Two players merely moved closely and a screen-play was not set but this play was classified as screen-play incorrectly because of the distance between players.

Fig 4d–4f show the trade-off between the recall and precision of the classifiers. We evaluated the F score which is the harmonic mean of recall and precision. The mean F scores of the on-ball, off-ball and joint classifiers were 0.782, 0.473, and 0.691 (on-ball: 0.608, off-ball: 0.784), respectively. The mean F scores of the conventional method [18] were 0.794, 0.514, and 0.671 (on-ball: 0.583, off-ball: 0.770), respectively. If the feature vectors did not include moving distance, the mean F scores were 0.776, 0.472, and 0.691 (on-ball: 0.605, off-ball: 0.786), and without geometric information, the mean F scores were 0.769, 0.436, and 0.703 (on-ball: 0.614, off-ball: 0.802), respectively. Overall, the AUC and F scores in our method outperformed those of the conventional method for off-ball screen-plays (joint classifier), whereas, for the on-ball screen-play classifier, only the AUC in our method was slightly better than that of the conventional method. Moreover, the AUC of the joint classifier outperformed that of the separate classifiers. Meanwhile, the F score of the separate classifiers was better than that of the joint classifier for the on-ball screen-plays, but that for off-ball screen shows the opposite results.

### Classification of detailed types of off-ball screen-plays

After detecting the on- and off-ball screen-plays, we additionally propose a more detailed classifier for off-ball screen-plays. In this study, 285 off-ball screen-plays were used and categorized into the seven types (Table 1). We used multi-class SVM, which has two strategies: one-against-all and one-against-one [31]. We primarily show the results of the one-against-all multi-class SVM (The result of the one-against-one approach is shown in S4 Fig). Note that the pin screen and flex screen did not have enough samples (pin: 23, flex: 8); thus they were eliminated from the analyses. The results show that the median AUC of down screen, flare screen, back screen, and cross screen were 0.883, 0.883, 0.897, and 0.923, respectively. The F scores were 0.722, 0.684, 0.476, and 0.706, respectively. Note that the number of each screen in the real game was 104, 68, 31, and 51 (Table 1).

Although our method achieved relatively higher performance than the conventional method, one of the limitations of the method is the difficulty in achieving perfectly accurate labelling (or definition) of the plays. Fig 4 shows examples of classification errors, where there was a false negative (miss) and false positive (false-alarm). In some actions of false negative, a screener set a screen-play but the user-defender avoided the screen-play and moved a relatively long distance (Fig 4a). In some actions of false positive, the screener stood on near the defenders but had no intention to set a screen-play (Fig 4b). It would be impossible to distinguish such plays from an actual screen-play using only a set of XY coordinates for each player. Both examples concerned the moving distance of the feature vectors, whereas the information on moving distance contributed to improving overall classification performance (Fig 3). Also among the detailed types of off-ball screen-plays, there were misclassifications due to the difficulty associated with accurately labelling (defining) using only the location data or videos (the details are explained in S1 Results and S5 Fig).

## Discussion

In this study, we first proposed a method to detect offensive cooperative plays, i.e., screen-plays at various situations and locations in actual basketball games. The proposed method accurately discriminated not only the cooperative plays in a primary area, i.e., near the ball, but also those distant from the primary area. We also proposed a method to classify the more detailed types of cooperative plays in various situations. This study contributes to understanding inconspicuous players to play important roles in team sports and could have the potential to detect well-defined and labeled cooperative behaviors. In this section, for each paragraph, we discuss the screen-play detection method (on- and off-ball screen-plays), classification of the detailed types of the off-ball screen-plays, the limitation of our findings, and the methodology of classification. We then follow up with conclusions.

In two-class classification (screen-play or not), our methods achieved higher performance in AUC (on-ball, 0.941; off-ball, 0.855) than that achieved using the previous feature vectors [18] that only classified on-ball screen-plays (on-ball: 0.933, off-ball: 0.825). Moreover, these values were greater than those obtained in the previous study [18] (AUC: 0.80–0.85), which may possibly be related to the difference in the segmentation system and the game data. Specifically, the moving distance of players and the geometric information, which was added to the feature vector of the previous study [18], improved classification performance. This indicates that these feature vectors capture the characteristics of play not only close to the ball, but also various types of plays at various locations on the court. If an appropriate amount of labeled cooperative behavior well-defined from location data can be obtained for agents and environments, the proposed framework (with a slight modification of the feature vector) might have the potential to classify various types of labeled cooperative plays for some sports and multi-agent systems. Note that the classification may work for local cooperative plays, such as screen-plays rather than global plays, such as team formations, because the latter often includes many players who are not involved in the global cooperation. Thus, classification for the latter would sometimes be an ill-defined problem.

Next, we additionally proposed the detailed off-ball screen-play classifier. The results for the off-ball screen-plays with multi-class SVM show better classification performance in off-ball screen-plays with a larger sample size. In this method, we assume a prior accurate off-ball screen-play classifier, because it might be difficult to directly detect detailed types of off-ball screen-plays. Off-ball screen-plays have more diversity in behaviors involving more than three players (passer, user and screener of the screen-play) [35], compared to the on-ball screen-plays, which may relatively depend on the individual skills of the player with the ball. This property of off-ball plays which may often occur with other team sports can make classification difficult; however, it should be essential to understand cooperative behavior in multi-agent. One possible improvement may be to increase the number of non-dominant types of screen-plays in training data in this study (Table 1); thus further study is required using larger samples to classify such non-dominant plays.

From the perspective of classification methods, we manually created feature vectors and used SVMs for classification. On the other hand, other approaches have attracted attention such as using an artificial neural network approach, which have been used to classify personal movements [36] without manual feature extraction. For team sports, this approach has classified broad movements of groups, such as team classification [37] and handclaps of spectators [38] at sports games. However, at present, we speculate that the approach using the hand-crafted feature may have advantages to discriminate the well-defined local cooperative plays, rather than the neural-network approach. This is possibly because the feature required for the task of this research might be considered to be the ability to classify the function as cooperative plays from the specific spatiotemporal relation of the positional data rather than extracting invariant and/or abstract structures. For cooperative plays in team sports, each player is not physically connected [39]; thus, the positional relation (i.e., the constraint condition) changes dynamically. These difficulties may also apply to other team sports (e.g., football) and more general multi-agent cooperative behavior; therefore it should be investigated further.

Overall, we derived accurate methods to detect cooperative plays using the moving distance of players, geometric information and the distance among the players, the goal, and the ball. Our method classified cooperative plays in cases both near to and distant from the ball. We also proposed a method to classify more detailed types of cooperative plays at various situations. Our framework may have the potential to detect well-defined labeled cooperative behavior. As an example of an application to sports, the system proposed in this study can visualize the movement of an offensive player who plays distant from the ball (off-ball plays). As a result, coaches and players may be motivated to focus more on off-ball plays, which are frequently inconspicuous, but effective.

## Supporting information

S1 Methods (DOCX)

S1 Results (DOCX)

S1 Fig Player movements and variation in various screen-plays. (a-h) Three representative examples of players and ball movements for the eight types of screen-plays. Symbols are the same as Fig 4. From a to h, they show down, flare, back, cross, pin, flex (off-ball), pick-and-roll, and hand-off (on-ball) screen-plays, respectively.
(TIF)

S2 Fig Signal, action and segmentation. The action interval is defined as the interval from 13 frames before and after the minimum distance between the candidate screener and user-defender during the signal.
(TIF)

S3 Fig Categorization of screen area and three-player angles. (a) The screen area is categorized into nine screener position areas. (b) The three-player angles are defined by the user (two candidates), screener and user-defender angles.
(TIF)

S4 Fig Confusion matrix of off-ball screen-play classification. This shows the results of off-ball screen-play classification. A row indicates correct play (visually classified) and a column indicates predicted play (classified by SVM). The diagonal elements represent correct classifications, and the other elements represent misclassifications.
(TIF)

S5 Fig Movement in correct and incorrect classifications. These images show examples of players and ball movement in correct and incorrect classifications. Symbols are the same as Fig 4. The left and center figures represent back screen and flare screen which were classified correctly. The right figure represents misclassification. This movement was classified visually as back screen but classified as flare screen by SVM.
(TIF)

S1 Movie The representative example of down screen. Red, magenta, blue and orange figure represent the screener, user, defense of user and the ball, respectively.
(MP4)

S2 Movie The representative example of flare screen. Explanation are the same as S1 Fig.
(MP4)

S3 Movie The representative example of pin screen. Explanation are the same as S1 Fig.
(MP4)

S4 Movie The representative example of back screen. Explanation are the same as S1 Fig.
(MP4)

S5 Movie The representative example of flex screen. Explanation are the same as S1 Fig.
(MP4)

S6 Movie The representative example of cross screen. Explanation are the same as S1 Fig.
(MP4)

S7 Movie The representative example of pick-and-roll screen. Explanation are the same as S1 Fig.
(MP4)

S8 Movie The representative example of hand-off screen. Explanation are the same as S1 Fig.
(MP4)
