# Supplementary material for: Automatically recognizing strategic cooperative behaviors in various situations of a team sport
Source: PLoS One. 2018 Dec 18;13(12):e0209247. doi: 10.1371/journal.pone.0209247 (PMC6298668; doi:10.1371/journal.pone.0209247)
Supplement: S1 Methods — (DOCX) [file pone.0209247.s001.docx]

# **S1 Methods**

**Measurements in a controlled experiment.** Each trial began at the initial position fixed by the coach with a pass from the defender to the dribbler, and the trial finished with a successful shot, rebound by a defender or turnover (i.e., out of bounds or defensive team holding the ball). In the event of a defensive foul or out of bounds, the offensive team started attacking again with the ball (i.e., a reset trial which was excluded from analysis). The game was played with two referees according to the rules of basketball, with the exception of the specific rules stated above.

Four reflective markers were placed on each player’s body (top and left of the head and on their right and left shoulders). We used the midpoint of shoulders as the representative point of the players. We also used a basketball pasted a reflective sheet in a striped pattern to improve ball handling and recorded its position. All raw coordinate data points were smoothed using a fourth-order Butterworth low-pass digital filter (6 Hz).

**Scene selection.** We constructed an automatic play-detection system using the positional data. First, we categorized the state of the ball as holding, passing and shooting. (1) If the ball and an attacker was near (horizontal distance was less than 1 m) and horizontal ball acceleration was less than –5 m/s^2^ during a ball catch (i.e., the ball was in the air), the state of the ball was defined as the holding. The involved attacker was defined as the ball attacker. (2) If the ball was not holding, ball vertical position was adequately above the ring (more than 3.2 m: the ring height was 3.05 m) and the ball was horizontally cloth to the ring (less than 0.5 m: the ring diameter was 0.45 m), the ball was detected as a shot. (3) If the ball was neither holding nor shot, the ball was passing because the trial was ended when a defender held the ball. We analysed the duration before an attacker’s shot or ball loss. When an attacker gained the ball after a shot (i.e., an offensive rebound), if the ball was brought out of the remote place from the ring (more than 5 m), we analysed the attack from the time to the shot.

**Support Vector Machine (SVM).** For the classification problem in this study, soft margin SVM with Gaussian-kernel was employed. SVM can separate data of two categories with constructing a hyper-plane to maximise the margin between positive and negative instances. The nearest data point from the hyper-plane is called support vectors. In SVM, this discriminant function is used:

$y=sign\left( \boldsymbol{w}^{t}\boldsymbol{x}-h \right),$ (1)

where $\boldsymbol{x}$ is the feature vector, $\boldsymbol{w}$ is a weight vector, $h$ is a scalar and $sign(x)$ represents the following sign function:

$sign\left( x \right)=\left\{ \begin{aligned} 1 :x>0 \\ -1 :x\leq0 \end{aligned}. \right.$ (2)

This means that SVM creates a set of feature vectors to separate two sets by a hyper-plane. In the training set, $\boldsymbol{w}$ is modulated and defined from training feature vector $\boldsymbol{x}$ and correct label $t$. $\boldsymbol{w}$ is required to set to separate two sets with best quality. Therefore, $\boldsymbol{w}$ is defined as the shortest distance between the separating hyper-plane and the vector closest to the hyper-plane, i.e., the ‘margin’. After all, we can replaces the problem to find a hyper-plane as the problem to find a parameter $\boldsymbol{w}$**.** Thus, the objective function is expressed as follows:

$L\left( \boldsymbol{w} \right)=\frac{1}{2}\left\| \boldsymbol{w} \right\|^{2},$ (3)

under the limiting condition where $i$ is the ordinal number:

$t_{i}\left( \boldsymbol{w}^{T}\boldsymbol{x}_{i}-h \right)\geq1, \left( i=1,\ldots,N \right).$ (4)

The soft margin SVM tolerates a degree of error such that the method of SVM is applied to a nonlinear distribution. The error is given by $\frac{\xi_{i}}{\left\| \boldsymbol{w} \right\|}$, where $\xi_{i}(\geq0)$ is a parameter, so that the object function changes where $\gamma$ is a penalty parameter :

$L\left( \boldsymbol{w},\boldsymbol{\xi} \right)=\frac{1}{2}\left\| \boldsymbol{w} \right\|^{2}+\gamma\sum_{i=1}^{N} \xi_{i} ,$ (5)

under the following limiting condition:

$\xi_{i}\geq0, t_{i}\left( \boldsymbol{w}^{T}\boldsymbol{x}_{i}-h \right)\geq1-\xi_{i} \left( i=1,\ldots,N \right).$ (6)

In addition, Gaussian-kernel ($\mathbf{K}$) makes the nonlinear transformation to help linear separation such that a separation can be formed even if it can not separate linearly:

$\mathbf{K}\left( \boldsymbol{x}_{1},\boldsymbol{x}_{2} \right)=\exp\left( \frac{-\left\| \boldsymbol{x}_{1}-\boldsymbol{x}_{2} \right\|^{2}}{2\sigma^{2}} \right).$ (7)

**Classification with one-against-one SVM.** We used the one-against-one SVM to classify the detailed types of off-ball screen-plays. The one-against-one method constructed *k*(*k*-1)/2 SVM models, where *k* is the number of classes. To distinguish the $i$th class and $j$th class, in the $i$th class is labelled positive, the $j$th class is labelled negative and all other classes are ignored. Here, creating feature vectors and training each SVM is the same as a conventional SVM. The output of a classifier given as $r_{ij}\in\left[ 0,1 \right]$ is the confidence of the binary classifier discriminating classes $i$ and $j$ in favour of the former class. The confidence of the classifier for the latter is computed by $r_{ji}=1-r_{ij}$ if the classifier did not provide it (the class with the largest confidence is the output class of a classifier).

After classification, each SVM votes for the predicted class. The votes for each class are counted, and the class with the greatest number of votes is regarded as predicted class, i.e.,

$Class=\arg\max_{i=1,\ldots,m} \sum_{1\leq j\neq i\leq m} s_{ij},$ (8)

where $s_{ij}$ is a vote for the classifier to distinguish the $i$th class and the $j$th class and $s_{ij}$ is 1 if $r_{ij}>r_{ji}$ and 0 otherwise.
