# Supplementary material for: Automatically recognizing strategic cooperative behaviors in various situations of a team sport
Source: PLoS One. 2018 Dec 18;13(12):e0209247. doi: 10.1371/journal.pone.0209247 (PMC6298668; doi:10.1371/journal.pone.0209247)
Supplement: S1 Results — (DOCX) [file pone.0209247.s002.docx]

**S1 Results**

**Off-ball screen-play classification using one-against-one multi-class SVM.** In addition to the one-against-all SVM, we also classified off-ball screen-play using the one-against-one SVM. S4 Fig shows the classification result. In this study, down screen, flare screen, back screen, and cross screen were used for classification. Down screen and cross screen were classified relatively precisely; however, flare screen was sometimes misclassified as back screen. It appears that it is common between flare screen and back screen because the screener sets the screen distant from the ring. In addition, the user sometimes moves toward the ring in flare screen and sometimes moves away from the passer and the ring in back screen (cf. Table 1).

**Correct and incorrect classifications for detailed types of off-ball screen-plays.** In the one-against-all SVM, there were some misclassifications. S5 Fig shows an example of correct and incorrect classifications. The left and center ones represent back and flare screens which were classified correctly. In the left one, the screener set the screen from the defender’s backside distant from the goal and the user used this screen to move toward the ring, which is the same as the back screen characteristics described in Table 1. In the center one, the screener set the screen distant from the basket ring, and the user used it to move away from the passer and the ring, which is the same as the flare screen characteristics also shown in Table 1. However, in the right one, the classification by SVM was flare screen; however, the visual classification result was back screen. Here, the screener set the screen from the defender’s backside distant from the goal, just like back screen. However, the user used it to move away from the passer and the ring, just like flare screen. In this study, it would be impossible to distinguish this situation from a real screen-play using only a set of XY coordinates for each player. Other parameters which represent the view of tactics (e.g. the direction of a player’s face) would be required.
