# Supplementary figures and images for: Automatically recognizing strategic cooperative behaviors in various situations of a team sport
Source: PLoS One. 2018 Dec 18;13(12):e0209247. doi: 10.1371/journal.pone.0209247 (PMC6298668; doi:10.1371/journal.pone.0209247)

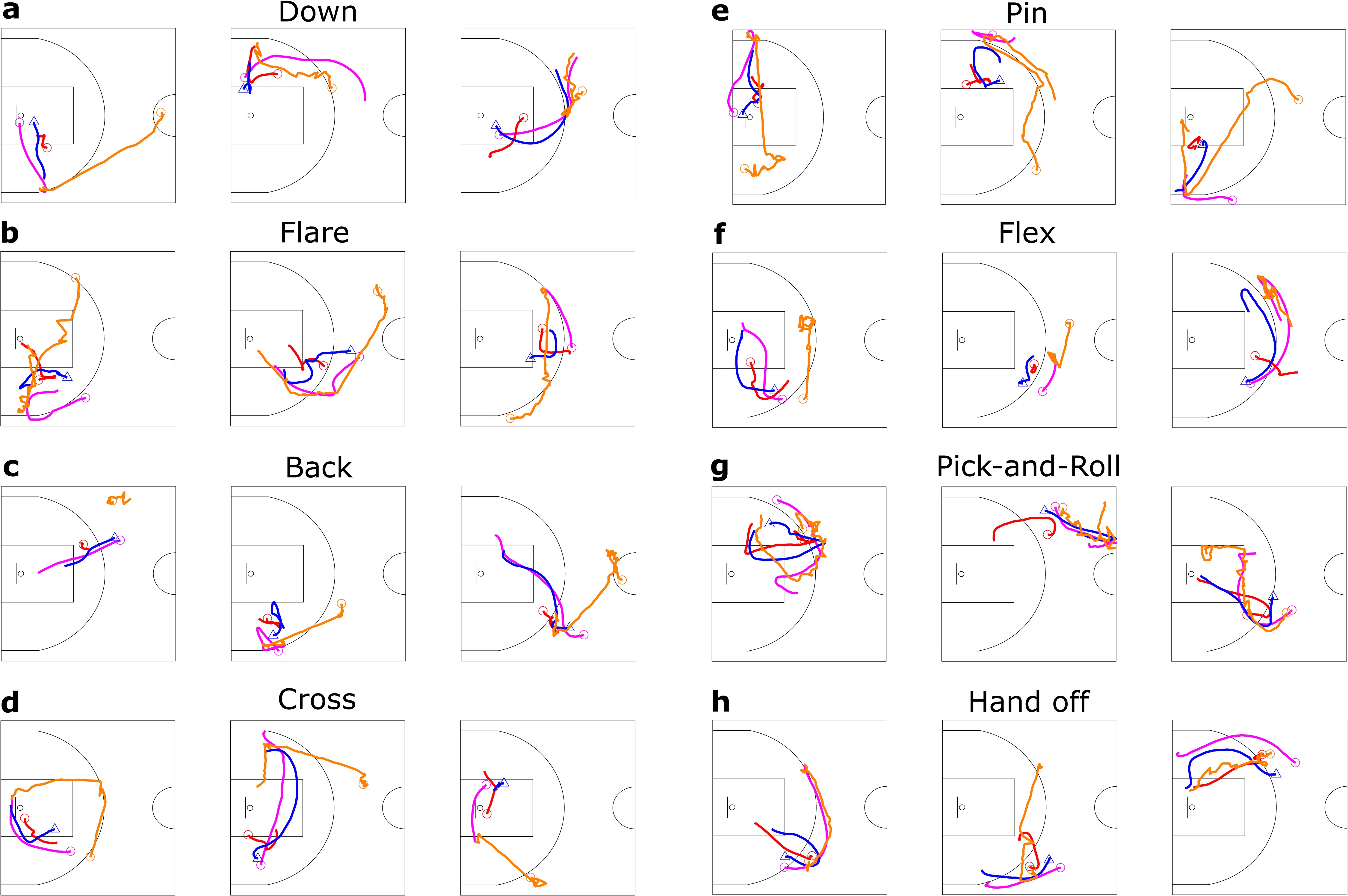

Supplement: S1 Fig — (a-h) Three representative examples of players and ball movements for the eight types of screen-plays. Symbols are the same as Fig 4. From a to h, they show down, flare, back, cross, pin, flex (off-ball), pick-and-roll, and hand-off (on-ball) screen-plays, respectively. (TIF) [file pone.0209247.s003.tif]

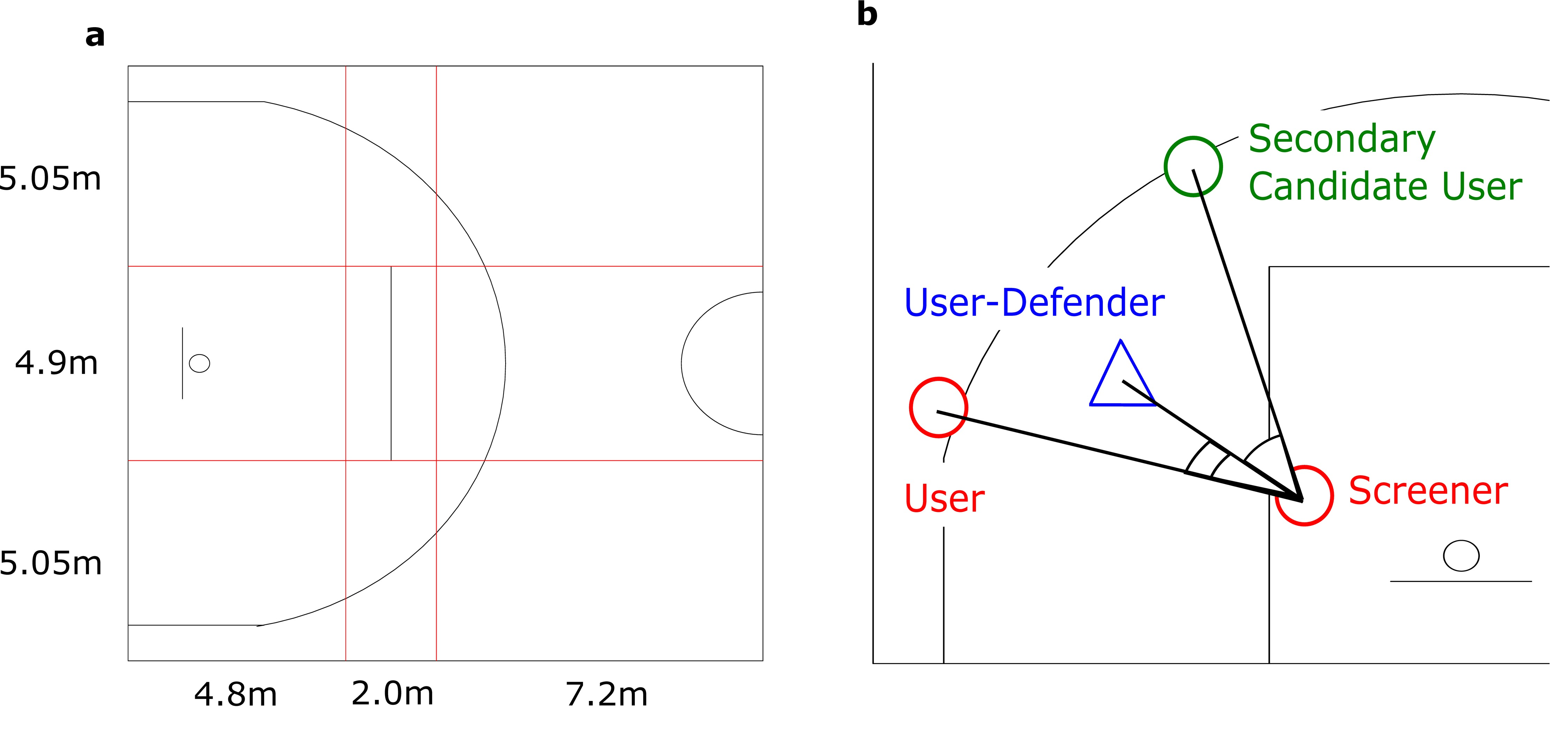

Supplement: S2 Fig — The action interval is defined as the interval from 13 frames before and after the minimum distance between the candidate screener and user-defender during the signal. (TIF) [file pone.0209247.s004.tif]

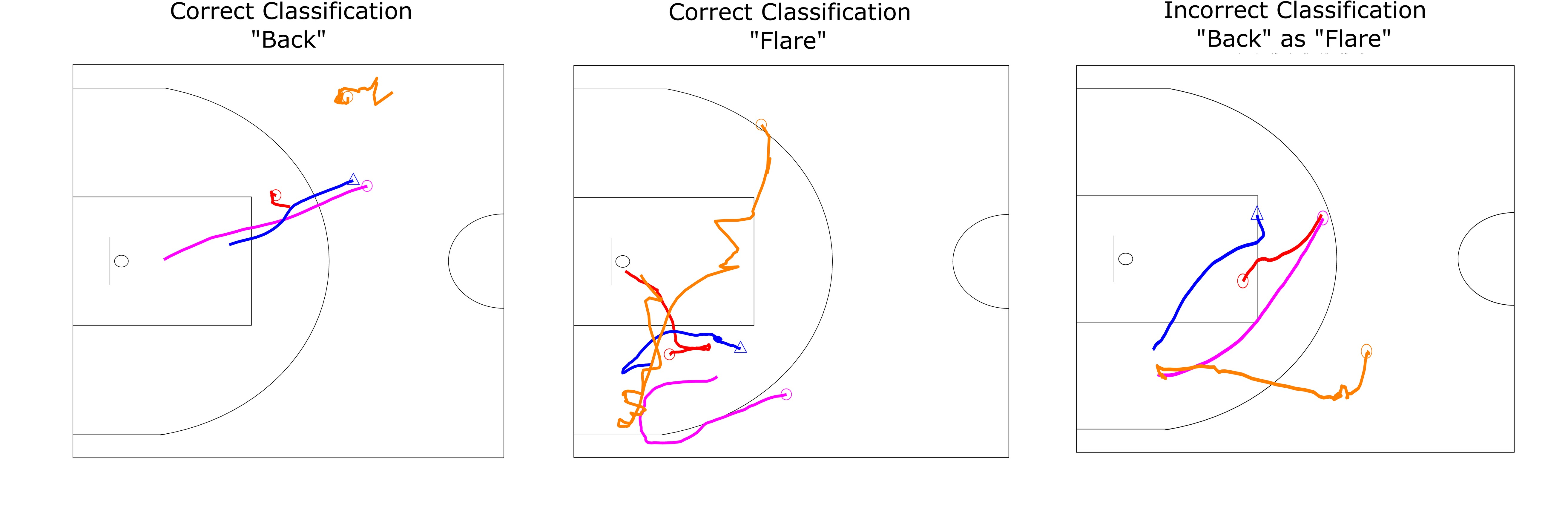

Supplement: S3 Fig — (a) The screen area is categorized into nine screener position areas. (b) The three-player angles are defined by the user (two candidates), screener and user-defender angles. (TIF) [file pone.0209247.s005.tif]

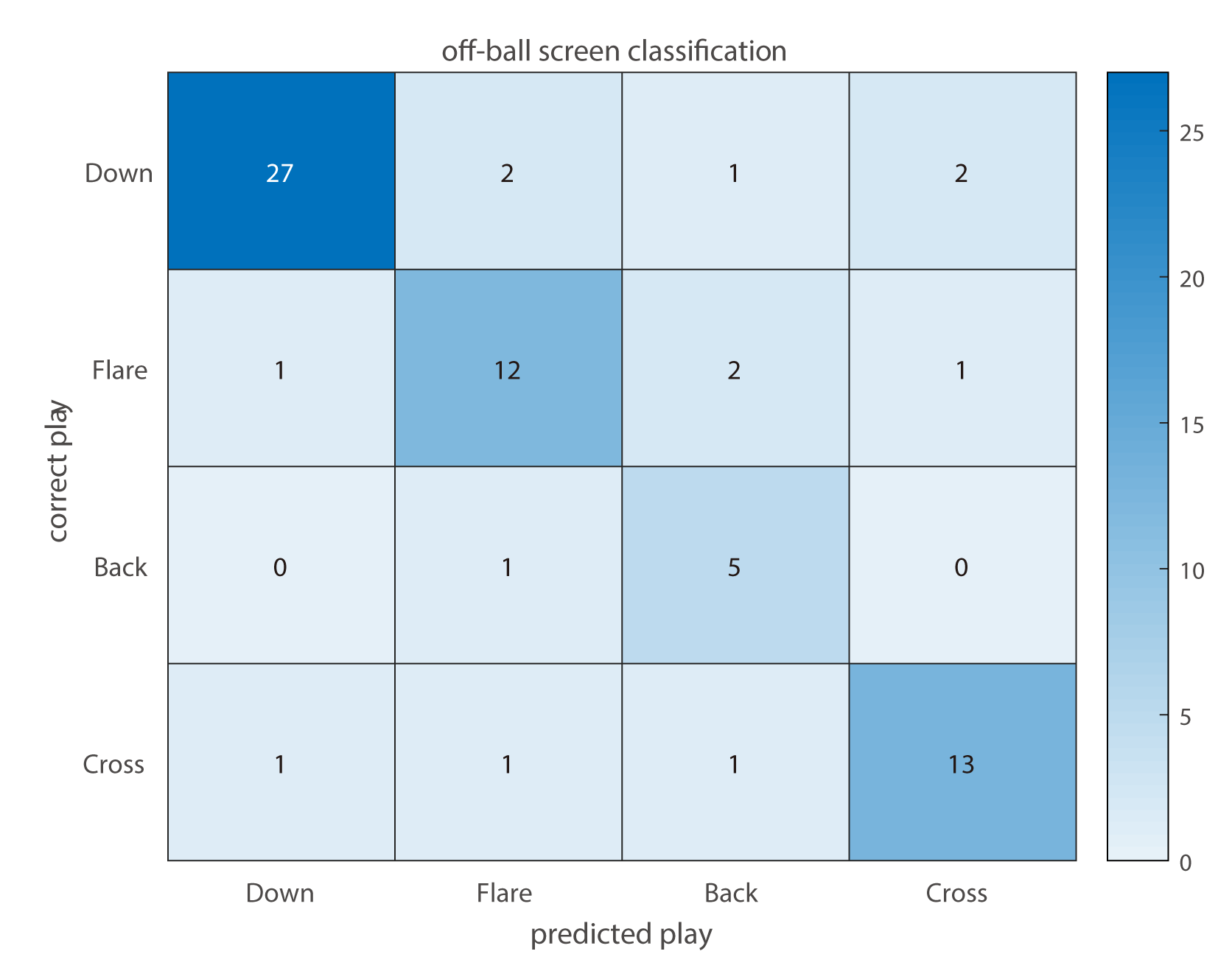

Supplement: S4 Fig — This shows the results of off-ball screen-play classification. A row indicates correct play (visually classified) and a column indicates predicted play (classified by SVM). The diagonal elements represent correct classifications, and the other elements represent misclassifications. (TIF) [file pone.0209247.s006.tif]

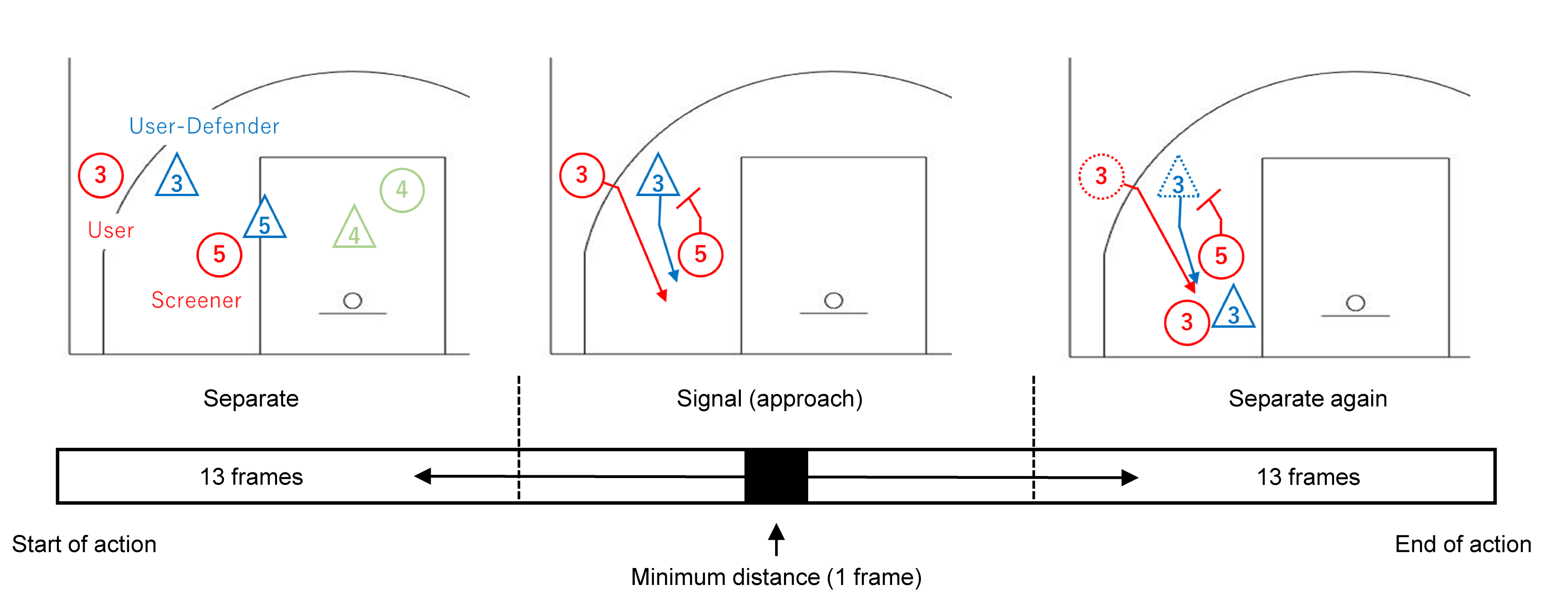

Supplement: S5 Fig — These images show examples of players and ball movement in correct and incorrect classifications. Symbols are the same as Fig 4. The left and center figures represent back screen and flare screen which were classified correctly. The right figure represents misclassification. This movement was classified visually as back screen but classified as flare screen by SVM. (TIF) [file pone.0209247.s007.tif]
